# Traditional Chinese Medicine Constitution Is Associated with the Frailty Status of Older Adults: A Cross-Sectional Study in the Community

**DOI:** 10.1155/2022/8345563

**Published:** 2022-05-25

**Authors:** Xingyu Ma, Hao Tang, Jinmei Zeng, Xueyan Pan, Xiaowen Luo, Jia Liao, Dongmei Liang, Lei Zhang, Shihao Zhou, Mingjuan Yin, Jindong Ni

**Affiliations:** ^1^Department of Epidemiology and Biostatistics, Dongguan Key Laboratory of Environmental Medicine, School of Public Health, Key Laboratory of Precision Public Health, Guangdong Medical University, Dongguan, 523808, Guangdong, China; ^2^Teaching & Research Department, Dongguan People's Guancheng Hospital, Dongguan, 523000, Guangdong, China

## Abstract

**Objective:**

This study explored the relationship between traditional Chinese medicine (TCM) constitution and frailty status in older adults.

**Methods:**

A total of 3,586 participants, 65 years of age and older, with complete data were evaluated. All received a complete frailty assessment and completed a TCM geriatric constitution questionnaire. Baseline characteristics and demographic information were collected. The relationship between the TCM constitution and frailty was evaluated by binary regression analysis. The consistency of the result was tested by multivariate linear regression.

**Results:**

The average prevalence of frailty among older adult participants was 12.5%. The three most prevalent biased constitutions in the frail older adult participants were phlegm dampness 140 (31.3%), Yin deficiency 77 (17.2%), and Yang deficiency 47 (10.5%). Univariate analysis showed that TCM constitution significantly correlated with frailty. After adjusting for potential confounders, binary logistic regression found a significant correlation between biased constitutions and frailty, including Qi stagnation (odds ratio (OR) = 3.51, 95% confidence interval (CI): 1.94–6.36)), Qi deficiency ((OR = 3.23, (95% CI: 1.76–5.94)), Yang deficiency ((OR = 2.37, (95% CI: 1.50–3.74)), phlegm dampness ((OR = 1.75, (95% CI: 1.24–2.48)), and Yin deficiency ((OR = 1.70, (95% CI: 1.15–2.50)). Results of multiple linear regression were consistent.

**Conclusions:**

TCM constitution was significantly associated with frailty status in older adults, and the distribution was different. Compared with a neutral constitution, older adults with Qi stagnation, Qi deficiency, Yang deficiency, phlegm dampness, and Yin deficiency were more likely to experience frailty.

## 1. Introduction

Populations are aging worldwide, and the trend will continue [1]. A recent estimate included a global increase in the proportion of people 65 years of age and older from 6.1% in 1990 to 8.8% in 2017 [2]. The rate of the increase will push the number of older adults to 1.6 billion by 2050 [3]. The size of the older adult population will pose considerable challenges for families, health-care systems, and public health policies [4, 5].

Aging is accompanied by the accumulation of health deficits [6], and frailty is a complex state of physical and psychological decline [7]. A decrease in physiological reserves is inevitable during the aging process. Susceptibility to disability increases, and aging, disease, and lack of physical activity all contribute to frailty [8]. We previously reported that sex, age, exercise frequency, education level, and other factors affect frailty subtypes [9]. A recent meta-analysis by Ofori-Asenso et al. reported that the pooled incidence of prefrailty was about 151/1000 person-years and that of frailty was 43/1000 person-years in community-dwelling older adults [10]. The prevalence and severity of frailty [9, 11] both increase with age [12, 13]. As older adults become frail, a downward spiral of physical conditions may be inevitable and can lead to falls, hospitalization, and death [7]. Frailty also leads to an increased risk of chronic diseases such as obesity [14] and hypertension [15].

In traditional Chinese medicine (TCM), a constitution is a relatively stable state of physical, psychological, and social adaptation formed by the interaction of congenital endowment and acquired accumulation and integration [16]. There are nine types of constitution, including one gentle constitution (neutral) and eight biased constitutions, which are Yin deficiency, Yang deficiency, phlegm dampness, damp heat, blood stasis, special diathesis, Qi stagnation, and Qi deficiency [16]. Bias is a response to an unbalanced state of the constitution. Previous studies have found that biased constitutions are associated with chronic noncommunicable diseases and that identification of TCM constitutions and the therapeutic effect of appropriate interventions improve the quality of life in older adults [17, 18].

Frailty is an old-age syndrome related to a variety of chronic diseases. In the theory of TCM, the constitution is an internal determinant of the occurrence, development, and prognosis of disease [19]. Identifying both disease and constitution is essential for the provision of personalized health care and rehabilitation programs to older adults. Of most interest is whether there is a correlation between the frailty status of older adults and their TCM constitution and whether there are differences in the frailty status associated with different TCM constitutions. This community-based study was designed to answer those questions. Clarifying these relationships will lay the foundation to identify and regulate the occurrence and development of frailty from the TCM perspective.

## 2. Materials and Methods

### 2.1. Older Adults

This annual study conducted free physical examination of residents over 65 years of age at the community at the Community Healthcare Center of Dalang Town, Dongguan City, Guangdong Province, from June 2020 to October 2020. We carried out the frailty assessment and TCM constitution identification during face-to-face interviews.

Men and women 65 years of age and older with the complete frailty assessment and TCM constitution assessment were eligible for inclusion. Older adults with Alzheimer's disease and those who were bedridden, could not communicate normally because of hearing and vision impairment, temporary residents (living for less than 5 years in the area), not willing to complete the assessment, or for whom the main TCM constitution could not be determined were excluded. Frailty and TCM constitution were evaluated in 3,900 older adults, the main constitution could not be determined in 314, and the remaining 3,586 were included in this study.

### 2.2. Survey Tools

1. A self-reported general status questionnaire collected age, sex, residence address, marital status, occupation, economic status, social participation (e.g., chess and card activities, membership in social organizations, and travel), lifestyle (e.g., exercise frequency), and other information.
2. Frailty assessment tools were based on the frailty index and included a self-developed frailty scale with 33 items in seven dimensions, FI = (sum of 33 item scores)/33. The index included general health status, the ability to perform daily life activities, functional activities, clinical symptoms and signs, cognitive function, social support, and mental state. This frailty index was used in our previous research on the aging population [9]. It has been recognized as reliable, sensitive, and effective for the determination and measurement of frailty in older adults. The optimal cutoff scores were 0.13 for early frailty and 0.22 for frailty.
3. Wang proposed the TCM constitution classification scale [16] that has been widely used in many studies of the association between constitution and obesity [20], diabetes [21], and other diseases. The TCM constitution questionnaire used in the community health-care center used the scale of “Classification and Determination of Traditional Chinese Medicine Constitutions for Older Adults” based on the original scale described by Wang and is more suitable for use with the characteristics of older adults. The improved scale has been adopted by the public health service system of the China Administration of Traditional Chinese Medicine [22], and it is also a tool for the identification of the traditional Chinese medicine system once a year for older adults aged 65 and above in the jurisdiction [23]. The classification consists of nine subscales including blood stasis, special diathesis, Yin deficiency, Yang deficiency, phlegm dampness, damp heat, Qi stagnation, Qi deficiency, and neutral constitution. A total of 33 items are assessed, with scores ranging from 1 to 5. A neutral constitution required a score of ≥17 points. Classification by the other eight constitutions required scores of ≥11 points for a “yes” assessment. Scores between 9 and 10 were reported as “tendency yes.” For ease of analysis, only those with “yes” scores of ≥11 were included in the constitution group. The constitution status of participants with scores that placed them in two or more constitution types was considered “mixed.” As the main constitution plays a leading role in the overall body state, participants with mixed constitutions were classified by the condition with the highest score.

### 2.3. Statistical Analysis

Completed questionnaires were coded and imported into an EpiData 3.1 database by trained personnel. SPSS 25.0 (IBM Corp Armonk, NY, USA) was used for the statistical analysis. Descriptive statistics were used to describe the basic TCM constitution and frailty characteristics of the survey population. The values of continuous variables that did not have a normal distribution were reported as the median and interquartile range (Q1–Q3). Categorical variables were expressed as frequencies, and between-group differences were compared by the chi-square test. After univariate analysis, only frailty-related variables with *P* < 0.05 were included in the multivariate model. The relationships between the nine TCM constitutions and frailty were determined by binary logistic regression analysis. The risk of frailty of the different TCM constitutions was estimated. The model was adjusted for potentially related factors such as age and sex to determine the correlations between TCM constitutions and frailty. Multivariate and binary logistic regressions were used to assess whether the results were consistent. Two-sided *P* values <0.05 were considered statistically significant. Odds ratios (ORs) and their 95% confidence interval (CI) were calculated to assess the relative risk of the constitution and the frailty status in these older adult participants.

## 3. Results

### 3.1. Demographic Characteristics

Table 1 summarizes the demographic characteristics of older adult participants who completed the survey. The median (Q1–Q3) age was 71 (67–76) years. The median (Q1–Q3) frailty index was 0.106 (0.061– 0.167), the maximum frailty index score was 0.894, and the minimum score was 0.000. In this older adult population, 58.8% were healthy, 12.5% were frail, and 28.7% in the prefrailty stage.

### 3.2. Distribution of the TCM Constitution and Risk Factors of the Frailty Status in Older Adults

A total of 3586 TCM constitution assessments were obtained in the study population. The biased constitution of the top three was phlegm dampness in 1145 (31.9%), Yin deficiency in 577 (16.1%), and blood stasis in 344 (9.6%) using a frailty index cutoff of 0.22, Among the frail older adults, the top three biased constitutions were phlegm dampness 140 (31.3%), Yin deficiency 77 (17.2%), and Yang deficiency 47 (10.5%). There were statistically significant differences in the distribution of TCM constitution and frailty state of older adults in different groups (*χ*^*2*^ = 100.421, *P* < 0.001).

Table 2 summarizes the results of univariate analysis of demographic characteristics, physical type, and frailty status of the study participants. Differences in the distribution of TCM constitutions in the frail participants and those who were not frail were significant. Differences in the demographic characteristics of the frail participants and those who were not frail were also significant (*P* < 0.001).

After adjusting for possibly related factors, binary logistic regression in Table 3 shows that Qi stagnation (OR = 3.51, *P* < 0.001), Qi deficiency (OR = 3.23, *P* < 0.001), Yang deficiency (OR = 2.37, *P* < 0.001), phlegm dampness (OR = 1.75, *P* < 0.001), and Yin deficiency (OR = 1.70, *P*=0.008) significantly increased the risk of frailty compared with the neutral constitution. The analysis also found that age was independently associated with a reduced risk of frailty. Compared with those ≥85 years of age, those who were 65–74 years of age had an OR of frailty of 0.22 and those who were 75–84 years of age had an OR of 0.46 (both *P* < 0.001). Male sex (OR = 0.75, *P*=0.037), living with relatives (OR = 0.55, *P* < 0.001), daily exercise (OR = 0.26, *P* < 0.001), and exercise at least once a week (OR = 0.46, *P*=0.026) were independently protective against frailty. Illiteracy was also associated with frailty (OR = 1.07, *P*=0.01) compared with junior high school and above.

Table 4 shows the results of multivariable logistic regression analysis of the TCM constitution and frailty of older adults, which were consistent with the binary logistic regression. Prefrail and frail were compared with the healthy status as in the control group. Neutral was the reference constitution, and Qi stagnation, Qi deficiency, Yang deficiency, phlegm dampness, and Yin deficiency were the risk factors for frailty and prefrailty (all *P* < 0.05). It was also found that blood stasis (OR = 1.41, 95% CI: 1.05–1.91) was independently associated with prefrailty.

## 4. Discussion

In this cross-sectional survey, we found an association between frailty status and TCM constitution in older adults. To the best of our knowledge, this is the first such community-based study to explore the relationship between TCM constitution and frailty status in the older adult population. We used a TCM constitution questionnaire that was designed for use in older adult populations [22]. The TCM constitution reflects the overall condition of older adults with a view of prevention first. Understanding that the association is crucial in both Western medicine and TCM. If the TCM constitution is known, then personalized interventions can improve the outcome of disease, improve the state of weakness, and ultimately achieve the goal of healthy aging [24].

We found that the prevalence of frailty among older adults in the community was 12.5% and that of prefrailty was 28.7%. In this study, frailty was considered as either nonfrail or frail. Our results are consistent with previous studies, which reported a prevalence of frailty among older adults in the community that generally exceeded 10% [25].

In this study, 84% of older adults were found to have a biased constitution. Other surveys have reported biased constitutions in more than 50% of the surveyed population [26], which is consistent with our results, but quite a few studies have reported less than 50% [27], possibly because different criteria were used in the selection of TCM constitutions. We found that frailty status was associated with different constitutions. The prevalence of frailty was relatively high in participants with Qi deficiency, Qi stagnation, and Yang deficiency. In ancient Chinese books, aging is accompanied by declines in Qi, blood, and Yang, weakened muscles, insufficient kidney essence, Yin, and Qi, weak viscera function, and lack of Qi, blood, and body fluid normal function. Therefore, the constitution of Yin and Yang deficiency and Qi deficiency and Qi depression is more common in older adults, which is in line with our research and some previous studies [28]. At the same time, environmental factors also affect the TCM constitution of older adults. The area selected for this study is in southern China, which has a subtropical monsoon climate. Mildew, rainy seasons, and high humidity are likely to result in dampness and heat evil [27], consistent with the increased prevalence of phlegm dampness in the study participants.

In this study, the frailty status varied within different bias constitutions. One interesting finding was that binary logistic regression of both nonfrail and frail participants and multivariate linear regression of healthy, prefrailty, and frail status both found that Qi stagnation, Qi deficiency, Yang deficiency, phlegm dampness, and Yin deficiency were independently associated with the risk of frailty in older adults. Frailty is a syndrome involving multiple organs and complex regulatory systems [29] that are involved in the occurrence, progression, and prognosis of chronic diseases such as cancer and cardiovascular disease. The disease prognosis is worse in frail than in healthy older adults [30, 31]. Some studies have found a significant association between chronic diseases and TCM constitution. For example, phlegm dampness, Yin deficiency, and Qi deficiency were found to be the three most common constitutions of people with hypertension, and phlegm dampness has been associated with cardiovascular risk factors [32, 33]. Previous studies have also found that older adults with Qi deficiency were more likely to have severe neurologic deficits than those with other constitutions [34]. Both Yin and Yang deficiency constitutions are associated with cold and heat evil. Older adults with Yang deficiency tend to be obese [20]. Obesity can cause oxidative stress, which can trigger cell aging, lead to local inflammation, and interfere with normal cellular repair and organization, which combine to lead to frailty [35].

Metabonomics studies have shown that older adults with Qi deficiency colon cancer are more likely to have severe metabolic disorders during the development of cancer [36]. Depression in TCM theory is caused by liver Qi stagnation, which is a mental disease caused by Qi stagnation [37]. Older adults with Qi deficiency, Yin and Yang deficiency, phlegm dampness, and Qi stagnation will have symptoms of decreased appetite and digestive function that may lead to vitamin D deficiency, thereby increasing the risk of fragility fractures [38], which would increase the probability of debilitation in older adults. All of the above indicate that biased constitutions are directly related to the risk and development of chronic diseases and provide evidence for its correlation with the degree and state of frailty.

Multivariate analysis showed that daily exercise and more than once weekly exercise were independently associated with protection from frailty. There is evidence that Tai Chi, an exercise closely associated with TCM, has an ameliorating effect on cardiovascular disease risk [39]. Exercise also counteracts the development of physical impairments associated with frailty in older adults [40]. The study results suggest that the lower risk of frailty in older adults with higher education levels might have been related to an increased focus on improved physical function and a relatively high level of medical literacy. As an alternative medicine, TCM improves the quality of life, alleviates chronic diseases, and alleviates the side effects of radiotherapy and chemotherapy [36, 41]. The constitution theory is based on choosing a treatment guided by individual variability. In TCM, classifying older adults with the same state of frailty by the constitution type would allow the establishment of personalized conditioning programs. Given the study results, additional in-depth investigations are recommended to explore the occurrence and development of frailty in people with different constitution types.

The study has some limitations. First, this study adopts a cross-sectional method to explore the relationship between TCM constitution and frailty status in older adults, but the TCM constitution types can be gradually adjusted. Therefore, it is necessary to carry out a longitudinal study to explain the time and causal effect of the change of TCM constitution in frail older adults. Second, in the study of multiple bias TCM constitutions, to accurately quantify the number of TCM constitutions of older adults, we only take the maximum value as the subject constitutions and do not analyze the constitutions with similar scores. Therefore, the combination and interaction between multiple constitutions and frailty outcomes, as well as between different constitutions, will be further explored in the future, to better achieve the purpose of early detection and early intervention.

## 5. Conclusions

TCM constitution was significantly related to frailty status in this older adult population, and the distribution of TCM constitutions differed in healthy, prefrailty, and frail participants. Compared with a neutral constitution, older adults with Qi stagnation, Qi deficiency, Yang deficiency, phlegm dampness, and Yin deficiency biased constitutions were more likely to become frail. The findings provide support for the benefit of the identification of TCM constitution in planning treatment to improve frailty status.

## Figures and Tables

**Table 1:** Characteristics of 3586 older adults.

Characteristics		*N*	%
Sex	Male	1424	39.7
	Female	2162	60.3

Age (years)	65–74	2531	70.6
	75–84	842	23.5
	≥85	213	5.9

Educational level^∗^	Illiteracy	747	20.8
	Primary school	2063	57.5
	Junior high school and above	772	21.5

Marital status^∗^	Married	2630	73.3
	Widowed/divorced/unmarried	951	26.5

Living condition^∗^	Living with nonrelatives	40	1.1
	Living with relatives	3058	85.3
	Living alone	486	13.6

Physical activity level	Everyday	3032	84.6
	Less than 5 times a week	63	1.8
	Less than 2 times a week	72	2.0
	No exercise	419	11.7

Frail condition	Healthy	2108	58.8
	Prefrail	1030	28.7
	Frail	448	12.5

*N* = 3586.  ^*∗*^Variables with missing values: educational level (4/3586; 0.1%), marital status (5/3586; 0.1%), and living condition (2/3586; 0.1%).

**Table 2:** Univariate analysis of frailty status, demographic characteristics, and TCM constitution in older adults.

		Frail level (n (%)) (*n* = 3586)		*P*
		Nonfrail (*n* = 3138)	Frail (*n* = 448)	
Sex	Male	1298 (41.4)	126 (28.1)	<0.001
	Female	1840 (58.6)	322 (71.9)	

Age (years)	65–74	2343 (74.7)	188 (42.0)	<0.001
	75–84	675 (21.5)	167 (37.3)	
	≥85	120 (3.8)	93 (20.8)	

Educational level	Illiteracy	559 (17.8)	188 (42.1)	<0.001
	Primary school	1853 (59.1)	210 (47.0)	
	Junior high school and above	723 (23.1)	49 (11.0)	

Marital status	Married	2393 (76.4)	237 (53.0)	<0.001
	Widowed/divorced/unmarried	741 (23.6)	210 (47.0)	

Living condition	Living with nonrelatives	15 (0.5)	25 (5.6)	<0.001
	Living with relatives	2763 (88.1)	295 (66.0)	
	Living alone	359 (11.4)	127 (28.4)	

Physical activity level	Everyday	2750 (87.6)	282 (62.9)	<0.001
	Less than 5 times a week	51 (1.6)	12 (2.7)	
	Less than 2 times a week	59 (1.9)	13 (2.9)	
	No exercise	278 (8.9)	141 (31.5)	

TCM constitution	Yin deficiency	500 (15.9)	77 (17.2)	<0.001
	Yang deficiency	206 (6.6)	47 (10.5)	
	Blood stasis	319 (10.2)	25 (5.6)	
	Special diathesis	75 (2.4)	12 (2.7)	
	Phlegm dampness	1005 (32.0)	140 (31.3)	
	Damp heat	268 (8.5)	31 (6.9)	
	Qi stagnation	64 (2.0)	29 (6.5)	
	Qi deficiency	56 (1.8)	30 (6.7)	
	Neutral	645 (20.6)	57 (12.7)	

**Table 3:** Binary logistic regression analysis on the TCM constitution and frailty of older adults.

Variables	*P* value	OR	95% CI
Age (years)			
65–74	<0.001	0.22	0.15–0.32
75–84	<0.001	0.46	0.32–0.67
≥85		1 (ref)	
Marital status			
Married	0.382	0.88	0.65–1.18
Widowed/divorced/unmarried		1 (ref)	
Educational level			
Illiteracy	0.001	2.07	1.38–3.09
Primary school	0.110	1.33	0.94–1.89
Junior high school and above		1 (ref)	
TCM constitution			
Yin deficiency	0.008	1.70	1.15–2.50
Yang deficiency	<0.001	2.37	1.50–3.74
Blood stasis	0.391	0.80	0.47–1.34
Special diathesis	0.137	1.74	0.84–3.62
Phlegm dampness	0.001	1.75	1.24–2.48
Damp heat	0.213	1.37	0.84–2.23
Qi stagnation	<0.001	3.51	1.94–6.36
Qi deficiency	<0.001	3.23	1.76–5.94
Neutral		1 (ref)	
Living condition			
Living with nonrelatives	0.097	2.01	0.88–4.57
Living with relatives	<0.001	0.55	0.40–0.76
Living alone		1 (ref)	
Physical activity level			
Everyday	<0.001	0.26	0.20–0.33
Less than 5 times a week	0.212	0.63	0.31–1.30
Less than 2 times a week	0.026	0.46	0.23–0.92
No exercise		1 (ref)	
Sex			
Male	0.037	0.75	0.58–0.98
Female		1 (ref)	

Abbreviations: OR, odds ratio; CI, confidence interval.

**Table 4:** Multiple logistic regression analysis on TCM constitution and frailty of older adults.

	Prefrail vs. healthy	Frail vs. healthy
Age (years)		
65–74	0.38 (0.26–0.57)^*∗∗*^	0.14 (0.09–0.21)^*∗∗*^
75–84	0.55 (0.36–0.82)^*∗∗*^	0.33 (0.22–0.51)^*∗∗*^
≥85	1 (ref)	1 (ref)
Sex		
Male	0.70 (0.59–0.83)^*∗∗*^	0.65 (0.49–0.86)^*∗∗*^
Female	1 (ref)	1 (ref)
Educational level		
Illiteracy	1.44 (1.09–1.89)^*∗∗*^	2.24 (1.49–3.38)^*∗∗*^
Primary school	1.27 (1.03–1.56)^*∗*^	1.36 (0.96–1.93)
Junior high school and above	1 (ref)	1 (ref)
TCM constitution		
Yin deficiency	1.38 (1.06–1.80)^*∗*^	1.95 (1.31–2.92)^*∗∗*^
Yang deficiency	2.20 (1.57–3.08)^*∗∗*^	3.42 (2.12–5.52)^*∗∗*^
Blood stasis	1.41 (1.05–1.91)^*∗*^	0.91 (0.53–1.55)
Special diathesis	1.22 (0.71–2.10)	1.89 (0.88–4.07)
Phlegm dampness	1.72 (1.38–2.16)^*∗∗*^	2.17 (1.52–3.11)^*∗∗*^
Damp heat	1.16 (0.84–1.61)	1.44 (0.87–2.39)
Qi stagnation	2.28 (1.34–3.89)^*∗∗*^	5.06 (2.64–9.68)^*∗∗*^
Qi deficiency	3.13 (1.78–5.53)^*∗∗*^	5.69 (2.86–11.33)^*∗∗*^
Neutral	1 (ref)	1 (ref)
Marital status		
Married	0.89 (0.72–1.10)	0.83 (0.61–1.12)
Widowed/divorced/unmarried	1 (ref)	1 (ref)
Living condition		
Living with nonrelatives	1.42 (0.48–4.19)	2.68 (0.91–7.90)
Living with relatives	0.66 (0.51–0.86)^*∗∗*^	0.45 (0.32–0.64)^*∗*^
Living alone	1 (ref)	1 (ref)
Physical activity level		
Everyday	0.60 (0.47–0.78)^*∗∗*^	0.20 (0.15–0.27)^*∗∗*^
Less than 5 times a week	0.47 (0.23–0.93)^*∗*^	0.46 (0.21–0.98)^*∗*^
Less than 2 times a week	0.69 (0.38–1.25)	0.38 (0.18–0.80)^*∗*^
No exercise	1 (ref)	1 (ref)

^*∗*^ *P* < 0.05; ^*∗∗*^*P* < 0.01.

## Data Availability

All data generated or analyzed during this study are included within the article.

## Supplementary Materials

Supplementary Materials The frailty scale for Chinese older adults (33 items).
