# Supplementary material for: Traditional Chinese Medicine Constitution Is Associated with the Frailty Status of Older Adults: A Cross-Sectional Study in the Community
Source: Evid Based Complement Alternat Med. 2022 May 25;2022:8345563. doi: 10.1155/2022/8345563 (PMC9159867; doi:10.1155/2022/8345563)
Supplement: Supplementary Materials — The frailty scale for Chinese older adults (33 items). [file 8345563.f1.docx]

**Frailty index for Chinese older adults**

| **General Health（6）** | |
| --- | --- |
| 1. Self-rating of health | 0=Good 0.5=Moderate 1=Poor |
| 2.How health has changed last year | 0=Better/Same 1=Worse |
| 3.How many times have been admitted to a hospital | 0=0 0.5=Once or two times  1=More than 2 times |
| 4.How many times (did you) fell last year | 0=0 0.5=Once 1=More than once |
| 5.BMI(Body mass index)  Height cm  Weight kg | 0=18.5≤BMI＜25  0.5= 25≤ BMI＜28  1= BMI <18.5 or BMI≥28 |
| 6.How many kinds of diseases are you Suffering from on a list of 19 diseases  Hypertension, Stroke, Cancer, Diabetes mellitus, Arthritis,  Chronic obstructive pulmonary disease, Cataract, Acute heart disease, Arrhythmia, Coronary heart disease, Chronic heart disease,  Tuberculosis, Gastrointestinal system disease, Chronic renal failure, Osteoporosis, Thyroid disease , Arteriosclerosis, Congestive heart failure, Parkinsonism | 0=0 0.5=One or two kinds 1=three or more |
| **Activities of daily living（9）:In the past 30 days, how much difficulty did you have in ….?** | |
| 7.Bathing | 0=No assistance 0.5= Some assistance  1= Needs assistance |
| 8.Dressing |  |
| 9.Eating |  |
| 10.Peparing meals |  |
| 11.Washing clothes by oneself |  |
| 12.Moving in house |  |
| 13.No-incontinence |  |
| 14.Using toilet |  |
| 15.Grooming |  |
| **Functional activities（6）: In the past 30 days, how much difficulty did you have in..?** | |
| 16.Up/Down stairs | 0=No assistance 0.5= Some assistance  1= Needs assistance |
| 17.Shopping |  |
| 18.Using the Telephone |  |
| 19.Walking long distance(1km) |  |
| 20.Going out with private or public transport |  |
| 21.Reaching and getting down a 1kg object from just above your head or bending down to pick up clothing from the floor |  |
| **Medical Symptoms（4）** | |
| 22.Bodily pain | 1= Most of time 0.5=Sometimes 0=Rarely |
| 23.Vision impairment | 1=Yes 0=No |
| 24. Hearing impairment | 1=Yes 0=No |
| 25.Having problems with sleeping | 1= Most of time 0.5=Sometimes 0=Rarely |
| **Psychological states（6）In last week, if you….?** | |
| 26.Feel everything is an effort | 1=Yes 0=No |
| 27.Feel depressed | 1=Most of time 0.5=Sometimes 0=Rarely |
| 28.Feel Lonely | 1=Yes 0=No |
| 29.Have problems with Memory | 1=Yes 0=No |
| 30.Have trouble get going | 1=Yes 0=No |
| 31.Feel full of energy | 1=No 0=Yes |
| **Social support（1）** | |
| 32.Live alone | 1= Yes 0= No |
| **Cognitive function （1）** | |
| 33.MMSE Score | 1=MMSE>17 0=MMSE≤17 |

**33. MMSE Scale （19）**

| 33.1.What year is this? | 1= Correct 0=Wrong |
| --- | --- |
| 33.2.What season is it now? | 1= Correct 0=Wrong |
| 33.3.What month is it? | 1= Correct 0=Wrong |
| 33.4.What's the date today? | 1= Correct 0=Wrong |
| 33.5.What day is today? | 1= Correct 0=Wrong |
| 33.6.What province are you in now? | 1= Correct 0=Wrong |
| 33.7.Which county (city) are you in now? | 1= Correct 0=Wrong |
| 33.8.What town(street) are you in now? | 1= Correct 0=Wrong |
| 33.9.Which floor are you on now? | 1= Correct 0=Wrong |
| 33.10.What is this place? | 1= Correct 0=Wrong |
| 33.1.Repeat (say): mask, clothes, cell phone | 3=all Correct 2=2 Correct 1=1 Correct 0=all Wrong |
| 33.12.Calculate 100-7 = □-7 = □-7 = □-7 = □-7 = □ | 5=all Correct 4=4 Correct 3=3 Correct 2=2 Correct 1=1Correct 0=all Wrong |
| 33.13.Recall 33.11 topic: masks, clothes, cell phones(question 33.11) | 3=all can be recalled 2=can recall 2 of them  1=can recall 1 of them 0=all Wrong |
| 33.14.Identification: watch, pencil | 2=all Correct 1=1Correct 0=all Wrong |
| 33.15.Repeat: Forty-four stone lions | 1= Correct 0=Wrong |
| 33.16.Follow the instructions: Take the paper in your right hand, fold it in half with both hands, and place it on your lap | 3=all Correct 2=2 Correct  1=1 Correct 0=all Wrong |
| 33.17."Close your eyes." Say the words and follow the instructions | 1= Correct 0=Wrong |
| 33.18.Please say a complete sentence | 1= Correct 0=Wrong |

33.19.Please draw it like this:

1=Two pentagons can be intersected and the intersecting graph is a quadrilateral 0=Failed to draw

FI = (Sum of 33 item scores)/33
